# High water turnover, hydration status, and heat stress among Daasanach pastoralists in a hot, semi-arid climate

**DOI:** 10.1093/emph/eoaf017

**Published:** 2025-07-18

**Authors:** Amanda McGrosky, Leslie Ford, Elena Hinz, Srishti Sadhir, Faith Wambua, David R Braun, Matthew Douglass, Emmanuel Ndiema, Rosemary Nzunza, Asher Y Rosinger, Herman Pontzer

**Affiliations:** Department of Biology, Elon University, Elon, NC, USA; Department of Evolutionary Anthropology, Duke University, Durham, NC, USA; Department of Biobehavioral Health, Pennsylvania State University, University Park, PA, USA; Department of Evolutionary Anthropology, Duke University, Durham, NC, USA; Department of Evolutionary Anthropology, Duke University, Durham, NC, USA; Department of Earth Sciences, National Museums of Kenya, Nairobi, Kenya; Center for the Advanced Study of Human Paleobiology, Anthropology Department, George Washington University, Washington, DC, USA; Technological Primate Research Group, Max Planck Institute for Evolutionary Anthropology, Leipzig, Germany; College of Agricultural Sciences and Natural Resources and Agricultural Research Division, University of Nebraska-Lincoln, Lincoln, NE, USA; Department of Earth Sciences, National Museums of Kenya, Nairobi, Kenya; Centre for Virus Research, Kenya Medical Research Institute, Nairobi, Kenya; Department of Biobehavioral Health, Pennsylvania State University, University Park, PA, USA; Department of Anthropology, Pennsylvania State University, State College, PA, USA; Department of Evolutionary Anthropology, Duke University, Durham, NC, USA; Duke Global Health Institute, Duke University, Durham, NC, USA

**Keywords:** water turnover, hydration, heat stress, temperature, kidney function

## Abstract

**Background and objectives:**

Water is essential for proper physiological function. As temperatures increase, populations may struggle to meet water needs despite adaptations or acclimation; chronic dehydration can cause kidney damage. We evaluate how daily water requirements are associated with ambient temperature (ambT), wet bulb globe temperature (WBGT), urine specific gravity (USG; marker of hydration status), and albumin:creatinine ratio (ACR; kidney function biomarker) among Daasanach pastoralists living in a hot, dry northern Kenyan climate.

**Methodology:**

Water turnover (WT), USG, and ACR were measured using deuterium depletion (WT), refractometry (USG), and urine dipstick (ACR) for 76 participants aged 5–68 years in June 2022–23. Relationships between WT, ambT, WBGT, USG, and ACR were evaluated using linear and generalized linear models.

**Results:**

Adult WT was higher than mean values worldwide, peaking around 7 l/day. Water demands increase from childhood through middle age before falling in later life. Adult WT was not correlated with ambT or WBGT. About 2/11 children’s and 7/36 adults’ USG indicated dehydration; USG was not correlated with child WT but was negatively correlated with adult WT when accounting for body size. WT was lower among adults with high (≥30 mg/g) ACR; high ACR was associated with higher USG.

**Conclusions and implications:**

High Daasanach WT is likely driven by hot, semi-arid conditions, and lifestyle, rather than by compromised kidney function. Most participants were well-hydrated. Despite nonsignificant correlations between temperature and adult WT, high WT highlights the physiological demands of hot, dry climates. As climate change increases the global population exposed to hotter temperatures, global water needs will likely increase.

## Introduction

Water is an essential human need. Human water needs vary by age, sex, reproductive status, body size, lifestyle (including activity level), climate, and environment [1–4]. The United States Institute of Medicine and European Food Safety Authority recommend that healthy, moderately active adults living in temperate climates consume between 2 and 3.7 l of water/day [2, 5, 6]; daily water requirements of adults living in extreme environments and/or with highly active lifestyles may top 8 l/day [2]. Here, we examine the daily water needs of a Daasanach community in northern Kenya, a physically active population living in a hot, semi-arid to arid environment.

The body uses water to transport nutrients and waste, give structure to tissues, protect against infection and disease, and regulate body temperature [7, 8]. Failure to meet water needs can lead to dehydration, heat exhaustion, kidney damage, cardiovascular failure, and ultimately, death [7–9]. Meeting daily water needs can be particularly challenging for those living in hot, dry climates with high levels of water insecurity [10]. As the planet warms, more people may face challenges meeting daily water needs. Hotter temperatures and heat waves not only increase the risk of dehydration and hyperthermia, but also decrease potable water availability. As temperatures climb, so does evaporation and water use [11]. These compounding factors may make it increasingly difficult for populations living in water-limited environments to obtain adequate supplies of water as water needs increase with ambient temperatures.

Daily water turnover (WT) represents the net total movement of water through the body per day and includes water lost through respiration, sweat, urine, and fecal material [4]. Work with adults living in an arid/semi-arid climate has identified high daily WT (i.e. high daily water requirements [12]) in conjunction with high water insecurity [13], but the relationship between daily WT, hydration status, and heat stress in this population, as well as how these metrics vary with age, is unclear. Evaluating the links between water needs, heat stress, and kidney health is necessary to understand the effects of climate change on water needs and human biology. Under conditions of extreme heat stress due to climate change, there is a growing concern that heat stress will negatively affect kidney health [14–16].

Heat stress and hydration status may be particularly relevant for populations living in extreme environments who have elevated water needs and who regularly experience water insecurity [17]. As physically active pastoralists living in a hot, semi-arid/arid environment with limited fresh water, Daasanach populations face both challenges. Although classic work from the 1960s suggests that populations indigenous to hot climates possess adaptations to heat stress that promote more efficient heat dissipation and thus lower sweat rates [18–20], water lost through sweat is only one component of total water needs. We have previously shown that Daasanach adults have high total water throughput [12]. Even in the presence of water-sparing adaptations to heat, a population’s water needs may be elevated by the quality of available water and challenges to kidney function. Past work with Daasanach communities has identified the use of moderately saline water sources, as well as hyper-dilute urine among participants [21, 22]. Because acute kidney injury, such as that caused by heat stress, hyperthermia, dehydration, malaria, and use of high-salinity water sources, can increase the risk of chronic kidney disease [23–26] and lead to a loss of the ability to maximally concentrate urine [27, 28], damaged kidneys need to excrete more water to eliminate waste. This may increase daily water throughput and individuals with chronic kidney disease may present with both high daily water needs and dilute urine.

To better understand water needs in a population indigenous to a hot climate who may be at risk for kidney disease, we evaluate how daily water requirements across the life course are associated with ambient weather conditions, heat stress, and hydration status among Daasanach communities. We test whether individuals with high WT and/or hyper-dilute urine exhibit albuminuria (albumin:creatinine ratios over 30 mg/g), which can be a sign of kidney disease. We use the results of these analyses to examine the impact of climate and water quality on water needs and kidney health, and to discuss the potential impact of future climate change on water needs and security among Daasanach and other populations living in hot, semi-arid/arid environments. As current climate models predict that up to one-third of the global population in 2050 could experience mean annual temperatures over 29°C [29], the current mean annual temperature experienced by Daasanach communities, billions of people may face similar challenges in the future.

## METHODOLOGY

### Daasanach pastoralists

About 48 000 Daasanach pastoralists live in Ethiopia and ~19 000 live in Kenya [30], with many members of the Kenyan group living in the vicinity of the town of Illeret (also spelled Ileret) in the Turkana Basin. The Turkana Basin has been one of the hottest places on earth for at least 3 million years and currently is in the top 1% hottest land areas in the world with a mean annual temperature of 29.2°C [31]. This region of the Greater Horn of Africa is highly susceptible to climate change and in recent years, drought, flash flooding, and climate variability have increased [32].

Traditional Daasanach lifestyles are physically demanding, with men tending to livestock and women bearing responsibility for water collection, cooking, childcare, and home maintenance [12, 33]. Communities located close to the main town of Illeret are more sedentary and market-integrated, with access to water standpipes, while those living in the *fora* (remote satellite grazing camps) practice a more traditional pastoralist lifestyle. For those living in the *fora*, obtaining water can be particularly time- and labor-intensive, as water is collected from hand-dug wells in dry riverbeds (“luggahs”) that can be 3–4 kilometers away from a community [13]. Fora communities typically drink more milk than more market-integrated communities, and many Daasanach communities have a daily tradition of drinking coffee and/or tea [22, 34, 35].

### Ethical approval and data collection

Ethical approval was obtained from The Pennsylvania State University Institutional Review Board (STUDY00009589), the Kenya Medical Research Institute (KEMRI/SERU/CVR/003/3739), the Director of Health in the county government of Marsabit, Kenya, and Daasanach community leaders. All adult participants and the parents/guardians of minor participants provided informed oral and written consent with the help of Daasanach language translators.

Participants were recruited to participate in June of 2022 and 2023 from communities in and around Illeret, Kenya. Illeret (4.314°N, 36.227°E) is located in Marsabit county on the arid/semi-arid northeastern shore of Lake Turkana. Illeret town and the surrounding region experience bimodal precipitation seasonality, with yearly rainfall averaging ~217 mm and temperatures ranging from 20°C to 37°C [36].

Eligible participants were individuals 5 years or older who resided in communities within or close to Illeret town. Body mass (weighed using a Tanita digital bioimpedance scale) and age (determined using a government ID or through estimation relative to major community events) were recorded for all 76 participants. About 45 participants were recruited in June 2022; 31 were recruited in June 2023.

Daily WT was measured using deuterium (^2^H) isotope depletion following established field sample collection protocols (e.g. Christopher et al. [37]). Each participant provided a baseline urine sample between 1 and 2 hours prior to consuming an ~5 g dose of deuterium (99.8% ^2^H_2_O) mixed with 80 g of potable water. At the time of baseline sample collection, ambient temperature and wet bulb globe temperature (WBGT, a measure of heat stress) were recorded from a Kestrel 5400 portable weather station for 47 participants (11 children, 36 adults). Urine specific gravity (USG), which measures the density of urine relative to the density of water and is a field-friendly urinary biomarker of hydration status, was measured in triplicate using a refractometer (Atago). The average USG value was used in analyses and a value greater than 1.020 was used as the cutoff point to signify hypohydration [38]. Albumin and creatinine were assessed using Germain Laboratories MicroAlbumin 2-1 Combo urine test strips on baseline urine samples. Albumin and creatinine data were available for 47 baseline urine samples (11 children, 36 adults) and were used to estimate albumin to creatinine ratio (ACR) and define albuminuria, which we classified as ACR ≥ 30 mg/g.

Additional urine samples were collected ~6 hours, 3–4 days, and 7 days after deuterium dose ingestion. Post-dose urine sample collection timing varied for some individuals due to logistical constraints (e.g. difficulty arranging transportation to remote collection sites or a participant traveling to the fora with their herds). All urine samples were stored in a field freezer before transfer to a −80°C freezer for storage prior to thawing and 30 kDa centrifuge filtering. Follow-up urine samples were not analyzed for USG or ACR.

Samples were analyzed via laser absorption mass spectrometry (ABB ICOS) for ^2^H enrichment. All samples were run at least three times; average isotope enrichments were used for subsequent calculations. WT (l/day) was calculated from ^2^H dilution space (ND) and elimination rate (kD) as 0.01802 × 1.043 × kD × ND/0.99, assuming 0.01802 l/mol H_2_O and a fractionation correction of 0.99 [39]. Total body water (TBW) was determined from isotope dilution (TBW = *N*_corr_ × 0.01802) and was used to calculate fat-free mass (FFM) assuming a hydration constant of 0.732 (FFM = TBW/0.732). Fat percentage was calculated by subtracting FFM from body mass and dividing by body mass.

### Analytical methods

Daily WT was modeled as a function of FFM, fat percentage, age, and/or sex for children (age < 18), adults (age ≥ 18), and all participants (age 5–68). Log-transforming FFM did not improve model performance and untransformed FFM did not violate regression model assumptions, so all models were run with untransformed FFM. Sex effects on the relationship between turnover and body size were tested by including sex as an interaction term in linear models. Adjusted WT was calculated by using a WT ~ FFM + sex linear model for all participants (age 5–68) to predict WT using each participant’s FFM and sex, subtracting predicted turnover from measured turnover, and converting to a percent. Absolute WT, adjusted WT, USG, and ACR were plotted against age to qualitatively assess patterns of WT, hydration, and kidney health across the life course. As changes in turnover with age in our sample were nonlinear, they were not modeled mathematically. Changes in USG with age were not modeled mathematically in our small sample as USG across the life course has been previously modeled in this population across a larger sample (see [40]).

The effects of ambient temperature and WBGT on WT were evaluated using multiple linear regression models of WT as a function of temperature metric (ambient or WBGT), sex, and/or FFM for both children (age < 18) and adults (age ≥ 18). As ambient and WBGT were only recorded at the time of initial urine collection, average daily maximum ambient temperatures and WBGTs were also calculated for each participant’s data collection week by pooling temperature data collected for all individuals throughout the data collection period.

WT was also modeled as a function of USG, ambient temperature, age, and/or sex for both adults and children using linear models or generalized linear models, as appropriate, to assess whether turnover was correlated with baseline hydration status. Differences in WT by hydration status were also evaluated by binning participants into hydrated (USG ≤ 1.020) or dehydrated (USG > 1.020) groups and testing for differences in absolute WT between hydrated and dehydrated individuals for both sexes using Mann–Whitney–Wilcoxon rank tests. Mann–Whitney–Wilcoxon tests were repeated on FFM and sex-adjusted WT between all hydrated and dehydrated adults.

To test whether high ACR and albuminuria (a potential indicator of kidney damage defined as albumin to creatinine ratios over 30 mg/g) were associated with higher WT in adult participants, individuals were classified as having either normal (<30 mg/g) or high (≥30 mg/g) ACR. WT was then modeled as a function of either this ACR binary, FFM, age, and/or sex. Differences in USG between adults with normal and high ACR were evaluated using Mann–Whitney–Wilcoxon rank tests.

All analyses were performed in R version 4.3.1; generalized linear models were run using the package *lme4* [41].

## RESULTS

### Turnover across body mass and life course

Mean WT summary statistics are presented in Table S1 by sex and age categories for the 76 participants. Absolute WT was highest for participants between age 18 and 40 and was higher among men (7.40 l/day) than women (6.90 l/day). WT was positively correlated with FFM across all participants (Table 1, Fig. S1). Age and body fat percentage were not significant predictors of WT in multiple linear regression models. There were no differences in the slope of the relationship between FFM and WT between the sexes across all ages and for the adult and children subgroups (Table 1; Table S2) and no effect of sex on WT for boys and girls (age < 18; Table S2). Being male, however, was negatively associated with WT in all-age models that included FFM (Table 1) and adult models that included FFM but not sex and/or age (Table S2).

**Table 1:** Multiple linear regression coefficient estimates for water turnover as a function of FFM, age, sex, fat percent, and/or temperature at baseline urine collection across participants; SE = standard error; *P* = *P* value.

All ages (*n* = 76)			Water turnover (l/day) ~															
Intercept			FFM (kg)			Fat %			Age (years)			Sex (M)			FFM × sex			Adj. *R*^2^
Est.	SE	*P*	Est.	SE	*P*	Est.	SE	*P*	Est.	SE	*P*	Est.	SE	*P*	*Est.*	*SE*	*P*	*Est.*
0.49	0.80	0.54	**0.15**	**0.02**	**<0.01**	0.02	0.02	0.40	–	–	–	–	–	–	–	–	–	0.53
0.20	1.08	0.85	**0.20**	**0.03**	**<0.01**	−0.03	0.03	0.28	–	–	–	1.11	1.23	0.37	−0.07	0.03	0.06	0.57
1.43	0.87	0.10	**0.16**	**0.02**	**<0.01**	−0.02	0.03	0.50	–	–	–	**−1.07**	**0.45**	**0.02**	–	–	–	0.56
0.93	0.58	0.11	**0.16**	**0.02**	**<0.01**	–	–	–	−0.01	0.01	0.52	**−0.87**	**0.36**	**0.02**	–	–	–	0.56
0.98	0.57	0.08	**0.16**	**0.02**	**<0.01**	–	–	–	–	–	–	**−0.89**	**0.36**	**0.02**	–	–	–	0.56
−0.33	0.86	0.73	**0.20**	**0.03**	**<0.01**	–	–	–	–	–	–	1.12	1.23	0.37	−0.06	0.04	0.09	0.57
0.88	0.60	0.14	**0.16**	**0.02**	**<0.01**	–	–	–	−0.01	0.01	0.44	–	–	–	–	–	–	0.53
Temperature models																		
Age < 18 (*n* = 11)			Water turnover (l/day) ~															
Intercept			FFM (kg)			WBGT (°C)			Ambient temp (°C)			Sex (M)			Sex × temp			Adj. R^2^
Est.	SE	*P*	Est.	SE	*P*	Est.	SE	*P*	Est.	SE	*P*	Est.	*SE*	*P*	*Est.*	*SE*	*P*	*Est.*
−5.25	5.13	0.33	–	–	–	0.34	0.20	0.11	–	–	–	–	–	–	–	–	–	0.17
−12.67	6.45	0.08	–	–	–	–	–	–	**0.52**	**0.20**	**0.03**	–	–	–	–	–	–	0.36
−6.40	8.89	0.50	–	–	–	0.38	0.34	0.30	–	–	–	2.36	11.76	0.85	−0.08	0.45	0.86	0
−17.32	9.23	0.10	–	–	–	–	–	–	0.65	0.29	0.06	9.27	13.81	0.52	−0.28	0.43	0.55	0.28
−0.19	4.49	0.97	**0.13**	**0.05**	**0.03**	0.05	0.19	0.81	–	–	–	–	–	–	–	–	–	0.49
−5.62	6.29	0.40	0.11	0.05	0.06	–	–	–	0.23	0.22	0.33	–	–	–	–	–	–	0.55
−0.12	4.79	0.98	**0.13**	**0.06**	**0.048**	0.05	0.21	0.83	–	–	–	−0.14	0.59	0.83	–	–	–	0.42
−5.83	7.15	0.44	0.10	0.06	0.11	–	–	–	0.23	0.24	0.37	0.05	0.59	0.94	–	–	–	0.48
−2.98	7.11	0.69	0.14	0.06	0.06	0.15	0.29	0.61	–	–	–	5.14	9.29	0.60	−0.20	0.36	0.59	0.36
−10.06	8.90	0.30	0.11	0.06	0.12	–	–	–	0.36	0.29	0.26	10.00	11.94	0.43	−0.31	0.38	0.44	0.46
−1.45	5.36	0.79	–	–	–	0.32	0.20	0.12	–	–	–	–	–	–	–	–	–	0.04
**11.92**	**5.77**	**0.046**	–	–	–	–	–	–	−0.15	0.18	0.41	–	–	–	–	–	–	0
−0.72	7.46	0.92	–	–	–	0.29	0.28	0.31	–	–	–	−1.79	11.09	0.87	0.07	0.41	0.86	0
12.38	7.77	0.12	–	–	–	–	–	–	−0.17	0.24	0.49	−1.16	12.06	0.92	0.04	0.38	0.91	0
−6.38	5.39	0.24	**0.12**	**0.004**	**0.02**	0.32	0.19	0.09	–	–	–	–	–	–	–	–	–	0.17
6.17	5.99	0.31	**0.11**	**0.05**	**0.03**	–	–	–	−0.12	0.17	0.49	–	–	–	–	–	–	0.10
−9.02	5.27	0.10	**0.22**	**0.06**	**<0.01**	0.30	0.18	0.10	–	–	–	**−1.81**	**0.84**	**0.04**	–	–	–	0.25
2.04	6.04	0.74	**0.22**	**0.07**	**<0.01**	–	–	–	−0.09	0.16	0.58	**−1.84**	**0.88**	**0.04**	–	–	–	0.19
−8.11	6.91	0.25	**0.23**	**0.07**	**0.03**	0.27	0.24	0.28	–	–	–	−3.80	9.72	0.70	0.08	0.36	0.84	0.22
1.61	7.70	0.84	**0.22**	**0.07**	**<0.01**	–	–	–	−0.08	0.22	0.72	−0.83	10.69	0.94	−0.03	0.33	0.93	0.16

Bolded values are significant at *p* < 0.05. “–” indicates variables not included in the model.

**Figure 1:**
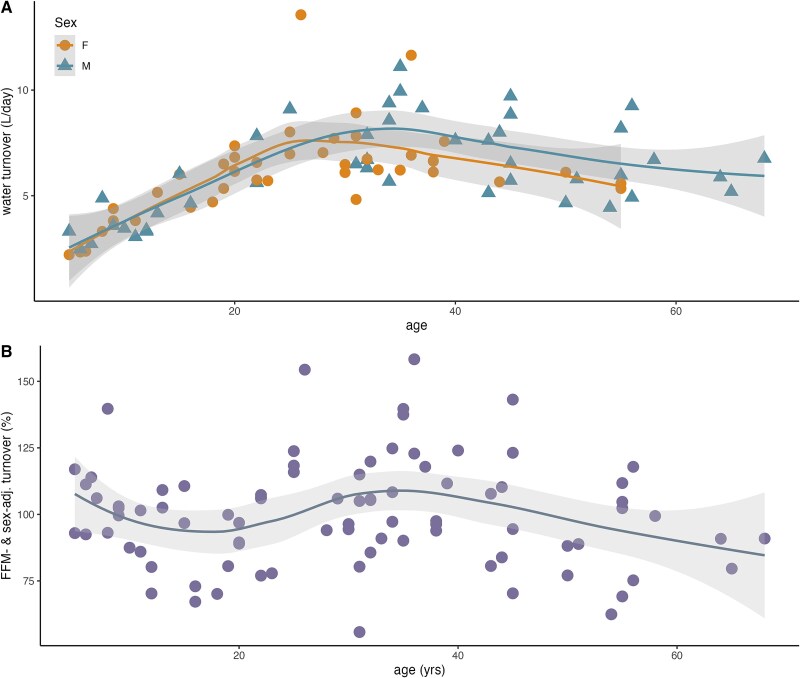
Absolute (A) and FFM and sex-adjusted (B) water turnover among Daasanach across the lifespan (*n* = 75), loess smooth.

While the relationship between WT and FFM, age, and/or sex was reasonably well fit by a linear model for participants under the age of 18 (*R*^2^ values between 0.52 and 0.60; Table S2), the relationship between WT and FFM, age, and/or sex was poorly explained by a linear model for adults (*R*^2^ values between 0.13 and 0.19; Table S2). Across the life course, absolute WT exhibits a nonlinear relationship with age, peaking before age 40 for both men and women before declining in middle and old age (Fig. 1A). FFM- and sex-adjusted WT was also nonlinear with age, with peaks early in life and in the late 30s (Fig. 1B).

### Ambient temperature and heat stress

**Figure 2:**
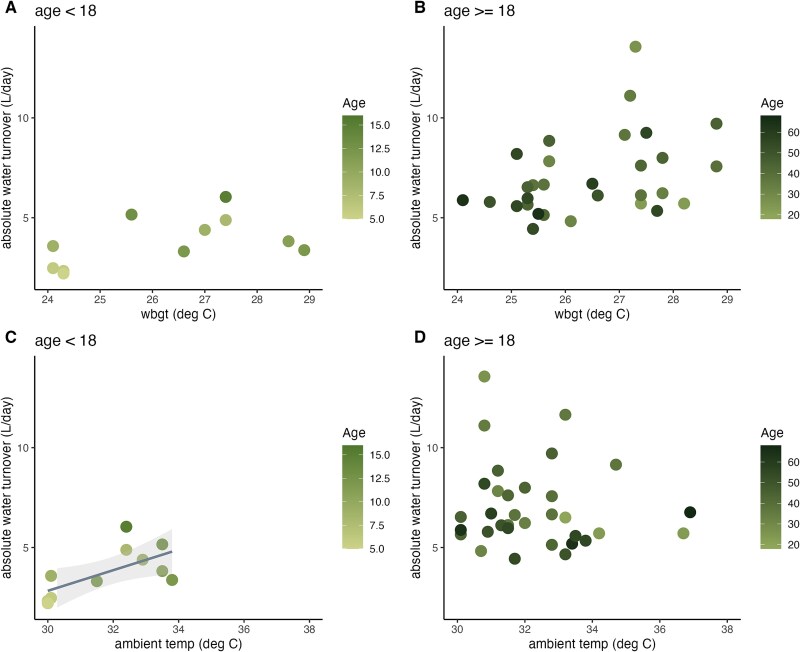
Absolute water turnover as a function of WBGT (A and B) and ambient temperature (C and D) at baseline urine collection for children (age < 18, *n* = 11) and adults (age ≥ 18, *n* = 36). Absolute turnover was significantly correlated with ambient temperature for children only (C) (adj. *R*^2^ = 0.36, *P* = .03).

Across the 11 children (age < 18) for whom temperature data were available, absolute WT was only correlated with ambient temperature (Fig. 2C), but not WBGT (Fig. 2A) when FFM was not included as a covariate (Table 1). When including FFM in models, neither WBGT (Fig. S2A) nor ambient temperature (Fig. S2C) were predictors of WT in children (Table 1). Absolute WT was not significantly correlated with either WBGT (Fig. 2B) or ambient temperature (Fig. 2D) in adults (*P* > .05; Table 1), including when accounting for FFM in multiple linear regression models (Table 1; Fig. S2B and D). Weekly average maximum ambient and WBGT were not significant predictors of WT in children or adults (Table S3).

### Hydration status

**Figure 3:**
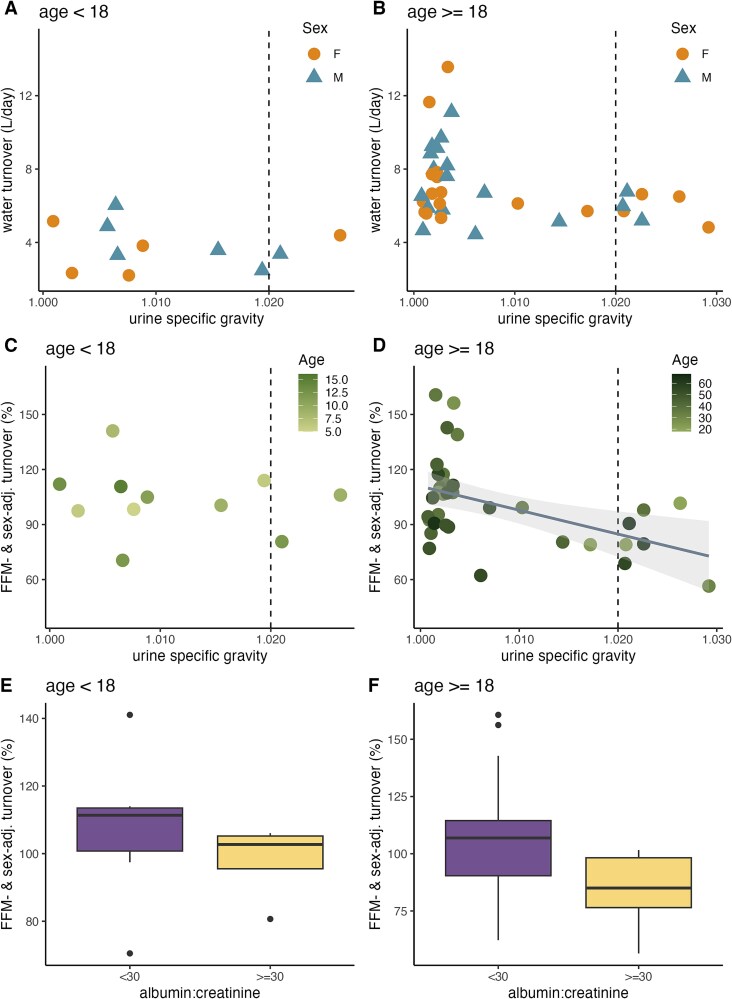
Absolute water turnover (A and B) and adjusted water turnover (C and D) are significantly correlated with USG in adults (*n* = 36) but not children (*n* = 11); dotted vertical line at 1.020 represents the cutoff for dehydration. Adjusted water turnover by ACR for adults (*n* = 36) (E) and children (*n* = 11) (F).

Across the sample of 11 children and 36 adults, 2/11 children and 7/36 adults had USG, a biomarker of hydrations status, over the “dehydrated” threshold of 1.020. USG has a nonlinear relationship with age, peaking early and late in life for male participants, but around age 20–30 for female participants (Fig. S3A). USG was not significantly correlated with WT among children in either single linear regression models of WT as a function of USG (Table 2; Fig. 3A) or multiple linear regression models of WT as a function of USG and FFM, ambient temperature, age, and/or sex (Table 2; Fig. 3C; Fig. S4A and C).

**Table 2:** Water turnover as a function of USG, lean mass, ambient temperature, age, and sex.

			Water turnover (l/day) ~																		
Intercept			FFM (kg)			USG			Ambient temp (°C)			Age (years)			Sex (M)			Sex × USG			Adj. *R*^2^
Est.	SE	*P*	Est.	SE	*P*	Est.	SE	*P*	Est.	SE	*P*	Est.	SE	*P*	Est.	SE	*P*	*Est.*	*SE*	*P*	*Est.*
Age < 18 (*n* = 11)																					
−9.56	49.16	0.85	0.12	0.05	0.06	10.76	48.85	0.83	–	–	–	–	–	–	62.86	89.70	0.51	−62.19	88.60	0.51	0.38
11.73	36.59	0.76	0.09	0.12	0.46	−10.71	36.07	0.78	–	–	–	0.09	0.26	0.74	–	–	–	–	–	–	0.43
13.73	36.76	0.72	0.12	0.13	0.37	−20.72	37.59	0.60	0.28	0.29	0.37	−0.07	0.31	0.84	–	–	–	–	–	–	0.43
15.79	37.91	0.69	0.09	0.06	0.20	−22.73	39.07	0.58	0.28	0.27	0.33	–	–	–	0.18	0.66	0.79	–	–	–	0.43
17.68	41.79	0.69	0.12	0.13	0.41	−26.15	44.26	0.58	0.34	0.36	0.39	−0.10	0.35	0.79	0.24	0.75	0.77	–	–	–	0.33
13.01	34.02	0.71	0.10	0.05	0.10	−19.11	34.24	0.59	0.25	0.23	0.31	–	–	–	–	–	–	–	–	–	0.51
12.66	34.43	0.73	**0.13**	**0.04**	**0.01**	−11.59	33.95	0.74	–	–	–	–	–	–	–	–	–	–	–	–	0.49
29.12	49.73	0.57	–	–	–	−25.07	49.18	0.62	–	–	–	–	–	–	–	–	–	–	–	–	0
Age ≥ 18 (*n* = 36)																					
**106.01**	**38.91**	**0.01**	**0.27**	**0.06**	**<0.01**	**−107.96**	**38.95**	**0.01**	–	–	–	–	–	–	18.51	63.70	0.77	−20.91	63.22	0.74	0.41
**111.52**	**30.71**	**<0.01**	**0.16**	**0.04**	**<0.01**	**−107.09**	**30.50**	**<0.01**	–	–	–	**−0.07**	**0.02**	**<0.01**	–	–	–	–	–	–	0.42
**118.62**	**32.13**	**<0.01**	**0.17**	**0.04**	**<0.01**	**−118.10**	**33.58**	**<0.01**	0.12	0.15	0.43	**−0.08**	**0.02**	**<0.01**	–	–	–	–	–	–	0.42
**123.09**	**32.02**	**<0.01**	**0.28**	**0.06**	**<0.01**	**−130.35**	**34.02**	**<0.01**	0.15	0.15	0.32	–	–	–	**−2.69**	**0.77**	**<0.01**	–	–	–	0.43
**126.19**	**31.45**	**<0.01**	**0.25**	**0.06**	**<0.01**	**−130.64**	**33.34**	**<0.01**	0.16	0.15	0.30	−0.04	0.03	0.14	−1.72	0.99	0.09	–	–	–	0.45
**96.10**	**36.17**	**0.01**	**0.13**	**0.05**	**0.01**	**−95.26**	**37.86**	**0.02**	0.05	0.17	0.77	–	–	–	–	–	–	–	–	–	0.23
**93.39**	**34.50**	**0.01**	**0.13**	**0.05**	**0.01**	**−90.88**	**34.26**	**0.01**	–	–	–	–	–	–	–	–	–	–	–	–	0.25
**90.08**	**37.78**	**0.02**	–	–	–	**−82.37**	**37.50**	**0.04**	–	–	–	–	–	–	–	–	–	–	–	–	0.10

Bolded values are significant at *p* < 0.05. “–” indicates variables not included in the model.

Among adults (age ≥ 18), there was a significant negative correlation between WT and USG (i.e. higher WT was associated with lower USG) in multiple linear regression models that included FFM as a predictor (Table 2; Fig. 3D), as well as in simple linear models of turnover as a function of USG alone (Table 2; Fig. 3B). In multiple regression models that included USG, age and being male were also negatively associated with WT (Table 2). When ambient temperature was added to adult multiple regression models (Table 2), USG remained negatively correlated with WT, but temperature was not a significant predictor of WT (Fig. S4B and D). As adult USG values clustered under 1.010, the relationship between WT and USG was also evaluated using generalized linear models with Gamma error structure and log link; significant predictors (Table S4) did not differ from multiple linear regression models.

Although ambient temperature (*R*^2^ = 0.48, *P* < .01) and WBGT (*R*^2^ = 0.06, *P* < .05) were positively correlated with urine collection time, there was only a statistically significant correlation between USG and collection time for adults (*R*^2^ = 0.01, *P* < .01), but not children (*P* = .35) or the full dataset (*P* = .07).

While the range of absolute WT was larger among hydrated (USG ≤ 1.020) adults than dehydrated (USG > 1.020) adults (Fig. S5A), Mann–Whitney tests revealed that absolute WT did not differ statistically between hydrated and dehydrated men (*W* = 35.5, *P* = .50; Fig. S5A) or women (*W* = 17.5, *P* = .29; Fig. S5A), or between hydrated and dehydrated male (*W* = 30, *P* = .48) and female participants (*W* = 35.5; *P* = .50) of all ages. FFM- and sex-adjusted WT, however, was higher among hydrated than dehydrated adults (*W* = 37.5, *P* = .01; Fig. S5B). As USG and WT were not significantly correlated in children, absolute WT was not compared between hydrated and dehydrated children.

### Albumin to creatinine ratio

Among all participants with ACR data (*n* = 38), albuminuria (ACR ≥ 30 mg/g) was associated with lower WT in multiple linear regression models that accounted for FFM, age, and/or sex (Table S5). Albuminuria (ACR ≥ 30 mg/g) was associated also with lower WT in adults in multiple linear regression models that accounted for FFM, age, and/or sex (Table S5; Fig. 3F). Albuminuria (ACR ≥ 30 mg/g) was not associated with WT in multiple linear regression models in children (Table S5; Fig. 3E). Absolute WT was negatively associated with albuminuria (ACR ≥ 30 mg/g) across all participants and among adults in simple linear models (Table S5). While ACR increased in men after age 50, we have no data for women at the oldest ages (Fig. S3); there was no significant effect of age or sex on ACR (Table S6) nor were there significant differences in ACR between males and females (*W* = 191, *P* = .98).

Mann–Whitney tests revealed that USG was significantly higher among both boys and men (*W* = 1, *P* < .01) and women and girls (*W* = 3, *P* < .01) with albuminuria (ACR ≥ 30 mg/g) than among those with normal ACRs (Fig. S6).

## DISCUSSION AND CONCLUSIONS

This paper highlights the high water demands of living in a hot, dry climate. WT among adult Daasanach participants was higher than mean values reported for a worldwide sample of adults (4.3 l/day men, 3.4 l/day women; [4]), peaking around 7 l/day prior to age 40. The trend of increasing water demands and then falling WT in later life is similar however to patterns observed in the US with daily water intake [42]. Trends in sex- and FFM-adjusted WT among Daasanach (Fig. 1B) have important implications for health and maintaining adequate hydration, as the high-size adjusted WT of children suggests that active children in a hot climate may be particularly vulnerable to dehydration. Indeed, USG is higher among Daasanach children than adults [40], a trend that appears in our subsample (Fig. S3A). If children cannot regularly meet water needs, they may face both short-term (e.g. hyperthermia) and long-term (e.g. kidney damage) adverse health outcomes.

Daasanach lifestyles are physically demanding and high levels of physical activity can lead to increased sweating and respiration, which can contribute to body water loss [12]. Human water needs can easily top 6–8 l/day with physical activity in hot, dry environments as moisture is lost to sweat [2]. While WBGT, a proxy for heat stress, was not correlated with WT in either adults or children and ambient temperature was only correlated with WT in children in this sample, this may be because there is not much variation in temperature metrics—it is simply always hot in June and July in northern Kenya. WBGT varied from 24°C to 30°C and ambient temperature from 30°C to 37°C, so it may be that temperatures did not dip low enough to relieve individuals from their high water needs. It also may be that our temperature and WT measurements are not nuanced enough to capture any heat-linked variations in WT, as ambient and WBGTs were only collected at baseline urine collection (a point measurement), while daily WT was measured over the course of 7 days. A larger sample size, with additional temperature data, could help to reveal whether WT is sensitive to temperature and heat stress in this population.

While these results are consistent with findings that high activity levels and high temperatures elevate water needs [2], they prompt a re-evaluation of the idea that populations indigenous to hot climates possess “water-sparing” adaptations that dramatically reduce water loss (e.g. [18, 19]). Despite the Turkana Basin of northern Kenya having been one of the hottest places on earth for millennia [31], Daasanach participants (who are presumably both adapted and acclimated to the local climate) nonetheless still face high water demands. Our results, however, do not negate the possibility that Daasanach participants are adapted to the Turkana Basin climate. Classic work on heat stress and water loss (e.g. [18–20]) focused on differences in sweat rate between individuals who were indigenous to a hot climate and those who were not; sweat rate is only one component of the total water throughput that we analyzed here.

In the absence of a comparative, nonindigenous population and sweat rate-specific data, we cannot evaluate whether Daasanach participants are more efficient sweaters or whether they have lower total WT rates than nonindigenous individuals in a similar climate. Prior work did not find that ancestry or the environment in which one spent their early life affected sweat gland density [43], but comparisons between Daasanach and non-locals would be a fruitful avenue for future research. We also note that although prior work found that individuals indigenous to a hot climate had lower sweat rates than nonindigenous individuals, sweat rates for both populations were still high: for example, mean sweat rates of acclimated individuals when performing 4 hours of work at WBGT of ~32°C were 428 ml/m^2^ hour among indigenous participants versus 478 ml/m^2^ hour among nonindigenous participants [19]. This result is consistent with our finding of elevated water needs in a hot climate, even when individuals may possess physiological adaptations to heat stress.

Despite potential challenges to obtaining water and higher than global average water requirements, participants in this study and the Daasanach community overall manage to meet their water needs. USG values suggest dehydration is relatively uncommon. Most participants (82% of children and 81% of adults) had USG values below the dehydrated threshold, indicating that many individuals are meeting their daily water needs. Deuterium-based methods do not directly measure water intake (which is ~80%–85% of WT volume [7]), but reducing mean adult Daasanach WT by 20% to calculate estimated intakes suggests that Daasanach adults consume (and therefore need to acquire) ~5.5–6 l of water per day. Maintaining adequate hydration is likely an economic and cultural priority; Daasanach have a daily habit of drinking tea, and water sharing is an integral component of Daasanach culture and a critical hydration strategy [21, 44].

While high WT can be related to reduced kidney function and chronic kidney disease [23–26], compromised kidney function does not appear to underpin the dilute urine and high WTs observed in Daasanach adults. We found that 36% (4/11) of participants under 18% and 19% (7/36) of participants between 18 and 58 had albuminuria (ACR ≥ 30 mg/g), a potential sign of kidney disease. An overall kidney disease incidence of 23% among those under 65 years old is high compared to US populations [45] and could be due to a high rate of false positives when using urine dipsticks, particularly for concentrated urine [46]. Dipstick positives require laboratory confirmation, but even if kidney disease is this common among the adult Daasanach population, we found no evidence that it increases WT. Adults with albuminuria had lower WT rates than adults with normal ACRs (Table S5, Fig. 3), suggesting that high WT is not associated with compromised kidney function (as assessed by ACR) in this population. Furthermore, although compromised kidney function was hypothesized to underpin hyper-dilute urine among Daasanach [22], participants with high ACRs had higher USG (i.e. more concentrated urine), indicating that elevated ACR was not associated with a decreased ability to concentrate urine. One direction for future work is to examine whether chronic dehydration in this population leads to elevated USG and then eventually to increased ACR and compromised kidney function (as assessed through glomerular filtration rate). Water salinity can also be linked to kidney health [22], but salinity was not examined here in relation to kidney function.

### Limitations, future directions, and implications for climate change

Only some of the sample (*n* = 47) in our dataset had both hydration and albumin:creatinine data, leading to small sample sizes, particularly for children (*n* = 11). Although WT rate did not differ between those with USG and ACR data and those without it (*P* > .05), it is possible that small sample sizes could skew the absence of a statistically significant association between WT and kidney function. We note, however, that high ACR was associated with lower WT rates, suggesting that despite the small sample, high WT among Daasanach participants is unlikely to be a consequence of compromised kidney function.

Additionally, ambient temperature and heat stress (WBGT) data were only available for the time of first urine collection for 47 participants, yet average daily WT was measured over a 7-day period. Temperature and WBGT fluctuate throughout the day, so the baseline measures used here are likely not indicative of the true total heat stress experienced by participants, which may further vary with sex, age, activity, and housing conditions. This study also only evaluated baseline hydration status on spot urine samples, but hydration is also dynamic [47] and likely varied over the 7-day period of WT measurement. Future work should build on this to incorporate more high-resolution heat stress and hydration data over time.

Ultimately, these results highlight that high WT rates among Daasanach are likely driven by the hot arid/semi-arid conditions and lifestyle rather than by kidney issues. These results also support prior work [12, 21] suggesting that individuals have adopted biocultural strategies to meet their water needs. Nonetheless, the high absolute WT among Daasanach participants and high FFM-adjusted turnover among children highlights the need for clean water access and infrastructure to ensure that water needs continue to be met to prevent adverse health outcomes such as hyperthermia, dehydration, and compromised kidney function. Despite the lack of significant correlation between temperature and heat stress and WT among adult participants, absolutely high WT rates in this population highlight the significant water needs of individuals living in a hot, semi-arid environment. Although the child sample was small and temperature effects disappeared when accounting for body size, the higher absolute WT with higher ambient temperature high FFM-adjusted turnover among Daasanach children suggests that children may be particularly vulnerable to dehydration and the physiological effects of increasing global temperature.

As climate change progresses and the global population living in hot and dry climates increases [29], global water demands will likely increase. Meeting these needs, as Daasanach participants do, is essential to help prevent chronic dehydration, kidney damage and comorbidities. To ensure that the global population is able to meet high climate- and lifestyle-driven water demands and mitigate the negative health consequences of chronic dehydration, it is essential to prioritize access to adequate potable water so that populations not only can continue to meet current water demands, but also have the resources to anticipate and address future increases in water needs.

## Supplementary Material

Supplemental_Information_eoaf017

## Data Availability

De-identified data that support this study are available from the authors at reasonable request.
