# Supplementary material for: High water turnover, hydration status, and heat stress among Daasanach pastoralists in a hot, semi-arid climate
Source: Evol Med Public Health. 2025 Jul 18;13(1):215–28. doi: 10.1093/emph/eoaf017 (PMC12409784; doi:10.1093/emph/eoaf017)
Supplement: Supplemental_Information_eoaf017 [file supplemental_information_eoaf017.docx]

**Supplemental Information**

**Table S1.** Water turnover (L/day), USG, body mass (kg), fat-free mass (kg), and height (m) summary statistics for Daasanach males (n=38; M) and females (n=38; F) stratified by age bins. Body surface area was calculated as $\surd(height\left( cm \right)*mass\left( kg \right)*3600)$, following (Mosteller 1987).

|  | **Water turnover (L/day)** | | **USG** | | **Body mass (kg)** | | **Lean mass (kg)** | | **Height (m)** | | **Body surface area (m^2^)** | |  | |
| --- | --- | --- | --- | --- | --- | --- | --- | --- | --- | --- | --- | --- | --- | --- |
|  | M | F | M | F | M | F | M | F | M | F | M | F | M | F |
| *age (yrs)* | *mean (sd)* | *mean (sd)* | *mean (sd)* | *mean (sd)* | *mean (sd)* | *mean (sd)* | *mean (sd)* | *mean (sd)* | *mean (sd)* | *mean (sd)* | *mean (sd)* | *mean (sd)* | *max n* | *max n* |
| 5-  9.9 | 3.40  (0.94) | 3.07 (0.91) | 1.014  (0.007) | 1.012 (0.013) | 18.2 (4.2) | 20.0 (5.0) | 15.3 (4.3) | 16.8 (4.5) | 1.16 (0.13) | 1.23 (0.12) | 0.76 (0.13) | 0.83 (0.14) | 5 | 6 |
| 10-13.9 | 3.48 (0.42) | 4.49 (0.94) | 1.014 (0.010) | 1.005 (0.006) | 27.4 (3.8) | 28.3 (3.3) | 23.8 (3.1) | 22.7 (3.5) | 1.43 (0.06) | 1.46 (0.03) | 1.04 (0.09) | 1.07 (0.07) | 5 | 2 |
| 14-17.9 | 5.34 (0.99) | 5.23 (1.10) | 1.006 (NA) | NA | 44.2 (4.7) | 44.4 (0.4) | 39.1 (7.5) | 32.5 (0.4) | 1.68 (0.04) | 1.60 (0.01) | 1.43 (0.09) | 1.41 (0.004) | 2 | 2 |
| 18-39.9 | 8.65 (1.56) | 7.10 (1.99) | 1.003 (0.001) | 1.011 (0.011) | 50.4 (1.9) | 42.0 (5.8) | 45.6 (2.1) | 32.1 (4.5) | 1.70  (NA) | 1.64 (0.04) | 1.55 (NA) | 1.47 (0.07) | 9 | 24 |
| 40+ | 6.73 (1.62) | 5.67 (0.32) | 1.007 (0.008) | 1.002 (0.001) | 53.8 (9.1) | 45.5 (4.6) | 44.7 (5.9) | 32.0 (4.7) | 1.73 (0.05) | 1.62 (0.07) | 1.63 (0.11) | 1.43 (0.10) | 17 | 4 |

**Table S2**. Multiple linear regression coefficient estimates for water turnover as a function of fat-free mass, age, sex, and/or fat percent for adults and children; SE = standard error; p = p-value.

| **age < 18** (n=22) | | | water turnover (L/day) ~ | | | | | | | | | | | | | | |  |
| --- | --- | --- | --- | --- | --- | --- | --- | --- | --- | --- | --- | --- | --- | --- | --- | --- | --- | --- |
| intercept | | | fat-free mass (kg) | | | fat % | | | age (yrs) | | | sex (M) | | | fat-free mass*sex | | | adj. R^2^ |
| *est.* | *SE* | *p* | *est.* | *SE* | *p* | *est.* | *SE* | *p* | *est.* | *SE* | *p* | *est.* | *SE* | *p* | *est.* | *SE* | *p* | *est.* |
| 0.57 | 0.78 | 0.48 | **0.16** | **0.03** | **0.00** | -0.01 | 0.03 | 0.74 | -- | -- | -- | 1.79 | 0.97 | 0.08 | -0.09 | 0.05 | 0.05 | 0.60 |
| 1.19 | 0.78 | 0.15 | **0.10** | **0.02** | **0.00** | 0.02 | 0.03 | 0.45 | -- | -- | -- | -0.09 | 0.36 | 0.80 | -- | -- | -- | 0.52 |
| **1.47** | **0.52** | **0.01** | 0.05 | 0.05 | 0.30 | -- | -- | -- | 0.12 | 0.12 | 0.33 | -0.18 | 0.33 | 0.58 | -- | -- | -- | 0.53 |
| **1.39** | **0.49** | **0.01** | 0.05 | 0.05 | 0.31 | -- | -- | -- | 0.12 | 0.12 | 0.30 | -- | -- | -- | -- | -- | -- | 0.55 |
| **1.67** | **0.48** | **0.00** | **0.10** | **0.02** | **0.00** | -- | -- | -- | -- | -- | -- | -0.20 | 0.33 | 0.54 | -- | -- | -- | 0.53 |
| **1.58** | **0.46** | **0.00** | **0.10** | **0.02** | **0.00** | -- | -- | -- | -- | -- | -- | -- | -- | -- | -- | -- | -- | 0.55 |
|  | | | | | | | | | | | | | | | | | | |
| **age ≥ 18** (n=54) | | | water turnover (L/day) ~ | | | | | | | | | | | | | | |  |
| intercept | | | fat-free mass (kg) | | | fat % | | | age (yrs) | | | sex (M) | | | fat-free mass*sex | | | adj. R^2^ |
| *est.* | *SE* | *p* | *est.* | *SE* | *p* | *est.* | *SE* | *p* | *est.* | *SE* | *p* | *est.* | *SE* | *p* | *est.* | *SE* | *p* | *est.* |
| 0.83 | 3.58 | 0.82 | **0.20** | **0.08** | **0.02** | -0.05 | 0.04 | 0.24 | -- | -- | -- | -0.17 | 4.75 | 0.97 | -0.04 | 0.11 | 0.72 | 0.18 |
| 1.76 | 2.45 | 0.48 | **0.18** | **0.05** | **<0.01** | -0.05 | 0.04 | 0.19 | -- | -- | -- | **-1.87** | **0.77** | **0.02** | -- | -- | -- | 0.19 |
| 1.15 | 2.58 | 0.66 | **0.17** | **0.06** | **0.01** | -- | -- | -- | -0.02 | 0.02 | 042 | -1.02 | 0.91 | 0.27 | -- | -- | -- | 0.18 |
| 3.49 | 1.52 | 0.02 | **0.12** | **0.04** | **<0.01** | -- | -- | -- | -0.03 | 0.02 | 0.06 | -- | -- | -- | -- | -- | -- | 0.17 |
| -0.17 | 2.01 | 0.93 | **0.19** | **0.05** | **<0.01** | -- | -- | -- | -- | -- | -- | **-1.48** | **0.72** | **0.045** | -- | -- | -- | 0.18 |
| 2.68 | 1.50 | 0.07 | **0.11** | **0.03** | **<0.01** | -- | -- | -- | -- | -- | -- | -- | -- | -- | -- | -- | -- | 0.13 |

**Table S3.** Linear regression coefficient estimates for water turnover as function of average maximum daily wet bulb globe temperature (avgWBGT), average maximum daily ambient temperature (avgAmb), over urine collection week and multiple linear regression coefficient estimates for water turnover as a function of average maximum temperatures, fat-free mass, sex, age, and/or urine specific gravity (USG); SE = standard error; p = p-value.

| **age < 18** (n=11) | | | water turnover (L/day) ~ | | | | | | | | | | | | | | | | | | |
| --- | --- | --- | --- | --- | --- | --- | --- | --- | --- | --- | --- | --- | --- | --- | --- | --- | --- | --- | --- | --- | --- |
| intercept | | | fat-free mass (kg) | | | avgWBGT  (deg C) | | | avgAmbT  (deg C) | | | sex (M) | | | age (yrs) | | | USG | | | adj. R^2^ |
| *est.* | *SE* | *p* | *est.* | *SE* | *p* | *est.* | *SE* | *p* | *est.* | *SE* | *p* | *est.* | *SE* | *p* | *est.* | *SE* | *p* | *est.* | *SE* | *p* | *est.* |
| -12.83 | 73.83 | 0.87 | 0.15 | 0.06 | 0.05 | 0.52 | 1.34 | 0.71 | -- | -- | -- | -0.08 | 0.65 | 0.91 | -- | -- | -- | -1.88 | 45.11 | 0.97 | 0.34 |
| -8.60 | 100.53 | 0.94 | 0.14 | 0.24 | 0.59 | 0.42 | 2.02 | 0.84 | -- | -- | -- | -0.08 | 0.72 | 0.92 | 0.03 | 0.43 | 0.95 | -3.14 | 52.52 | 0.96 | 0.21 |
| -37.09 | 63.78 | 0.58 | 0.12 | 0.71 | 0.06 | -- | -- | -- | 1.37 | 1.45 | 0.38 | 0.44 | 0.71 | 0.56 | -- | -- | -- | -10.41 | 37.65 | 0.79 | 0.41 |
| -42.48 | 69.40 | 0.57 | 0.06 | 0.14 | 0.71 | -- | -- | -- | 1.48 | 1.58 | 0.39 | -0.47 | 0.77 | 0.57 | 0.13 | 0.29 | 0.66 | -9.17 | 40.49 | 0.83 | 0.33 |
| -47.51 | 47.94 | 0.35 | **0.12** | **0.05** | **0.04** | -- | -- | -- | 1.37 | 1.35 | 0.35 | -0.48 | 0.65 | 0.48 | -- | -- | -- | -- | -- | -- | 0.49 |
| -51.78 | 51.30 | 0.35 | 0.06 | 0.13 | 0.69 | -- | -- | -- | 1.48 | 1.45 | 0.34 | -0.51 | 0.69 | 0.49 | 0.14 | 0.26 | 0.62 | -- | -- | -- | 0.43 |
| -15.53 | 33.05 | 0.65 | **0.15** | **0.05** | **0.02** | 0.55 | 1.10 | 0.63 | -- | -- | -- | -0.08 | 0.60 | 0.90 | -- | -- | -- | -- | -- | -- | 0.44 |
| -13.80 | 46.20 | 0.78 | 0.14 | 0.20 | 0.49 | 0.49 | 1.54 | 0.76 | -- | -- | -- | -0.09 | 0.65 | 0.90 | 0.02 | 0.37 | 0.96 | -- | -- | -- | 0.34 |
|  | | | | | | | | | | | | | | | | | | | | | |
| **age ≥ 18** (n=36) | | | water turnover (L/day) ~ | | | | | | | | | | | | | | | | | | |
| intercept | | | fat-free mass (kg) | | | avgWBGT  (deg C) | | | avgAmbT  (deg C) | | | sex (M) | | | age (yrs) | | | USG | | | adj. R^2^ |
| *est.* | *SE* | *p* | *est.* | *SE* | *p* | *est.* | *SE* | *p* | *est.* | *SE* | *p* | *est.* | *SE* | *p* | *est.* | *SE* | *p* | *est.* | *SE* | *p* | *est.* |
| **95.78** | **39.95** | **0.02** | **0.27** | **0.06** | **<0.01** | 0.34 | 0.44 | 0.45 | -- | -- | -- | **-2.54** | **0.76** | **<0.01** | -- | -- | -- | **-107.89** | **32.70** | **<0.01** | 0.42 |
| **116.48** | **41.97** | **<0.01** | **0.23** | **0.06** | **<0.01** | 0.002 | 0.51 | 0.99 | -- | -- | -- | -1.58 | 1.07 | 0.15 | -0.04 | 0.03 | 0.22 | **-115.64** | **32.98** | **<0.01** | 0.43 |
| 87.98 | 44.38 | 0.06 | **0.26** | **0.06** | **<0.01** | -- | -- | -- | 0.80 | 0.99 | 0.43 | **-2.38** | **0.79** | **<0.01** | -- | -- | -- | **-118.35** | **31.20** | **<0.01** | 0.42 |
| 97.56 | 44.33 | 0.04 | **0.23** | **0.06** | **<0.01** | -- | -- | -- | 0.58 | 0.98 | 0.56 | -1.52 | 1.00 | 0.14 | -0.04 | 0.03 | 0.18 | **-11768** | **30.78** | **<0.01** | 0.44 |
| -25.30 | 14.93 | 0.10 | **0.22** | **0.07** | **<0.01** | 0.79 | 0.48 | 0.11 | -- | -- | -- | **-1.96** | **0.85** | **0.02** | -- | -- | -- | -- | -- | -- | 0.24 |
| -19.58 | 18.66 | 0.30 | **0.21** | **0.07** | **<0.01** | 0.65 | 0.56 | 0.25 | -- | -- | -- | -1.48 | 1.25 | 0.25 | -0.02 | 0.04 | 0.61 | -- | -- | -- | 0.22 |
| -4.43 | 43.48 | 0.92 | **0.18** | **0.07** | **0.02** | -- | -- | -- | 0.17 | 1.17 | 0.88 | -0.89 | 1.19 | 0.46 | -0.04 | 0.03 | 0.24 | -- | -- | -- | 0.19 |
| -15.07 | 41.78 | 0.72 | **0.22** | **0.07** | **<0.01** | -- | -- | -- | 0.39 | 1.17 | 0.74 | -1.79 | 0.92 | 0.06 | -- | -- | -- | -- | -- | -- | 0.18 |

**Table S4.** Adult (age >= 18) water turnover as a function of USG, lean mass, ambient temperature, age, and sex; generalized linear model (Gamma error structure, log link).

| **age ≥ 18** (n=36) | | | water turnover (L/day) ~ | | | | | | | | | | | | | | | | | |  |
| --- | --- | --- | --- | --- | --- | --- | --- | --- | --- | --- | --- | --- | --- | --- | --- | --- | --- | --- | --- | --- | --- |
| intercept | | | fat-free mass (kg) | | | USG | | | ambient temp (deg C) | | | age (yrs) | | | sex (M) | | | sex*USG | | | AIC |
| *est.* | *SE* | *p* | *est.* | *SE* | *p* | *est.* | *est.* | *p* | *est.* | *SE* | *p* | *est.* | *SE* | *p* | *est.* | *SE* | *p* | *est.* | *SE* | *p* | *est.* |
| **14.49** | **5.11** | **<0.01** | -- | -- | -- | **-12.44** | **5.07** | **0.02** | -- | -- | -- | -- | -- | -- | -- | -- | -- | -- | -- | -- | 145.31 |
| **14.20** | **4.83** | **<0.01** | **0.02** | **0.01** | **<0.01** | **-13.04** | **5.05** | **0.02** | 0.01 | 0.02 | 0.81 | -- | -- | -- | -- | -- | -- | -- | -- | -- | 141.08 |
| **13.90** | **4.60** | **<0.01** | **0.02** | **0.01** | **<0.01** | **-12.56** | **4.58** | **<0.01** | -- | -- | -- | -- | -- | -- | -- | -- | -- | -- | -- | -- | 139.16 |
| **14.27** | **4.95** | **<0.01** | **0.04** | **0.01** | **<0.01** | **-13.52** | **4.96** | **0.01** | -- | -- | -- | -- | -- | -- | 3.30 | 8.11 | 0.69 | -3.60 | 8.05 | 0.66 | 131.42 |
| **16.73** | **4.10** | **<0.01** | **0.02** | **0.01** | **<0.01** | **-15.68** | **4.29** | **<0.01** | 0.02 | 0.02 | 0.39 | **-0.01** | **0.003** | **<0.01** | -- | -- | -- | -- | -- | -- | 131.07 |
| **16.68** | **4.10** | **<0.01** | **0.04** | **0.01** | **<0.01** | **-16.52** | **4.35** | **<0.01** | 0.02 | 0.02 | 0.36 | **--** | **--** | **--** | **-0.35** | **0.10** | **<0.01** | -- | -- | -- | 130.68 |
| **15.71** | **3.96** | **<0.01** | **0.02** | **0.01** | **<0.01** | **-14.12** | **3.94** | **<0.01** | -- | -- | -- | **-0.01** | **0.003** | **<0.01** | -- | -- | -- | -- | -- | -- | 129.95 |

**Table S5.** Water turnover as a function of albumin to creatinine ratio (ACR) as a binary categorical variable of ACR <30 or ACR ≥ 30 (ACR ≥ 30 can indicate albuminuria).

| **All participants** (n=47) | | | water turnover (L/day) ~ | | | | | | | | | | | |  |
| --- | --- | --- | --- | --- | --- | --- | --- | --- | --- | --- | --- | --- | --- | --- | --- |
| intercept | | | fat-free mass (kg) | | | ACR ≥ 30 | | | age (yrs) | | | sex (M) | | | adj. R^2^ |
| *est.* | *SE* | *p* | *est.* | *SE* | *p* | *est.* | *SE* | *p* | *est.* | *SE* | *p* | *est.* | *SE* | *p* | *est.* |
| 1.32 | 0.88 | 0.14 | **0.20** | **0.03** | **<0.01** | **-1.46** | **0.53** | **<0.01** | -0.04 | 0.02 | 0.06 | **-1.17** | **0.48** | **0.02** | 0.56 |
| 1.29 | 0.93 | 0.17 | **0.19** | **0.03** | **<0.01** | **-1.35** | **0.55** | **0.02** | **-0.04** | **0.02** | **0.046** | -- | -- | -- | 0.50 |
| 1.57 | 0.90 | 0.09 | **0.16** | **0.02** | **<0.01** | **-1.36** | **0.54** | **0.02** | -- | -- | -- | **-1.24** | **0.49** | **0.02** | 0.53 |
| 1.57 | 0.95 | 0.11 | **0.14** | **0.02** | **<0.01** | **-1.22** | **0.57** | **0.04** | -- | -- | -- | -- | -- | -- | 0.47 |
| **6.81** | **0.38** | **<0.01** | -- | -- | -- | **-1.57** | **0.74** | **0.04** | -- | -- | -- | -- | -- | -- | 0.07 |
|  | | | | | | | | | | | | | | | |
| **Adults**  (n=36) | | | water turnover (L/day) ~ | | | | | | | | | | | |  |
| intercept | | | fat-free mass (kg) | | | ACR ≥ 30 | | | age (yrs) | | | sex (M) | | | adj. R^2^ |
| *est.* | *SE* | *p* | *est.* | *SE* | *p* | *est.* | *SE* | *p* | *est.* | *SE* | *p* | *est.* | *SE* | *p* | *est.* |
| **0.18** | **3.07** | **0.05** | **0.23** | **0.07** | **<0.01** | **-1.93** | **0.69** | **<0.01** | -0.03 | 0.03 | 0.35 | -1.74 | 1.09 | 0.12 | 0.36 |
| **4.07** | **1.90** | **0.04** | **0.15** | **0.04** | **<0.01** | **-1.63** | **0.68** | **<0.01** | **-0.06** | **0.02** | **0.01** | -- | -- | -- | 0.32 |
| -1.82 | 2.24 | 0.42 | **0.26** | **0.06** | **<0.01** | **-2.02** | **0.68** | **<0.01** | -- | -- | -- | **-2.44** | **0.80** | **<0.01** | 0.36 |
| 2.42 | 1.96 | 0.23 | **0.12** | **0.05** | **0.01** | **-1.53** | **0.74** | **0.045** | -- | -- | -- | -- | -- | -- | 0.20 |
| 7.41 | **0.37** | **<0.01** | -- | -- | -- | **-1.44** | **0.79** | **0.08** | -- | -- | -- | -- | -- | -- | 0.06 |
|  | | | | | | | | | | | | | | | |
| **Children**  (n=11) | | | water turnover (L/day) ~ | | | | | | | | | | | |  |
| intercept | | | fat-free mass (kg) | | | ACR ≥ 30 | | | age (yrs) | | | sex (M) | | | adj. R^2^ |
| *est.* | *SE* | *p* | *est.* | *SE* | *p* | *est.* | *SE* | *p* | *est.* | *SE* | *p* | *est.* | *SE* | *p* | *est.* |
| 1.37 | 1.30 | 0.34 | 0.12 | 0.15 | 0.46 | -0.32 | 0.69 | 0.66 | 0.03 | 0.33 | 0.92 | -0.30 | 0.71 | 0.67 | 0.20 |
| 1.24 | 1.17 | 0.33 | 0.10 | 0.13 | 0.46 | -0.27 | 0.64 | 068 | 0.05 | 0.30 | 0.87 | -- | -- | -- | 0.31 |
| 1.42 | **1.13** | 0.27 | **0.12** | **0.05** | **0.04** | -0.323 | 0.63 | 0.63 | -- | -- | -- | -0.31 | 0.64 | 0.64 | 0.33 |
| 1.29 | 1.04 | 0.25 | **0.13** | **0.04** | **0.03** | -0.26 | 0.59 | 0.67 | -- | -- | -- | -- | -- | -- | 0.41 |
| 4.03 | **0.51** | **<0.01** | -- | -- | -- | -0.24 | 0.80 | 0.77 | -- | -- | -- | -- | -- | -- | 0 |

**Table S6.** Non-binary ACR as a function of age and sex.

| **All participants** (n=47) | | | ACR (mg/g) ~ | | | | | | | | |  |
| --- | --- | --- | --- | --- | --- | --- | --- | --- | --- | --- | --- | --- |
| intercept | | | Age (yrs) | | | Sex (M) | | | Sex*Age | | | adj. R^2^ |
| *est.* | *SE* | *p* | *est.* | *SE* | *p* | *est.* | *SE* | *p* | *est.* | *SE* | *p* | *est.* |
| **47.27** | **16.14** | **0.01** | -0.65 | 0.48 | 0.18 | -12.74 | 21.71 | 0.56 | 0.48 | 0.58 | 0.41 | 0 |
| **37.95** | **10.15** | **0.01** | -0.29 | 0.25 | 0.26 | -- | -- | -- | -- | -- | -- | 0 |
| **37.13** | **10.48** | **0.01** | -0.32 | 0.27 | 0.24 | 3.47 | 9.34 | 0.71 | -- | -- | -- | 0 |


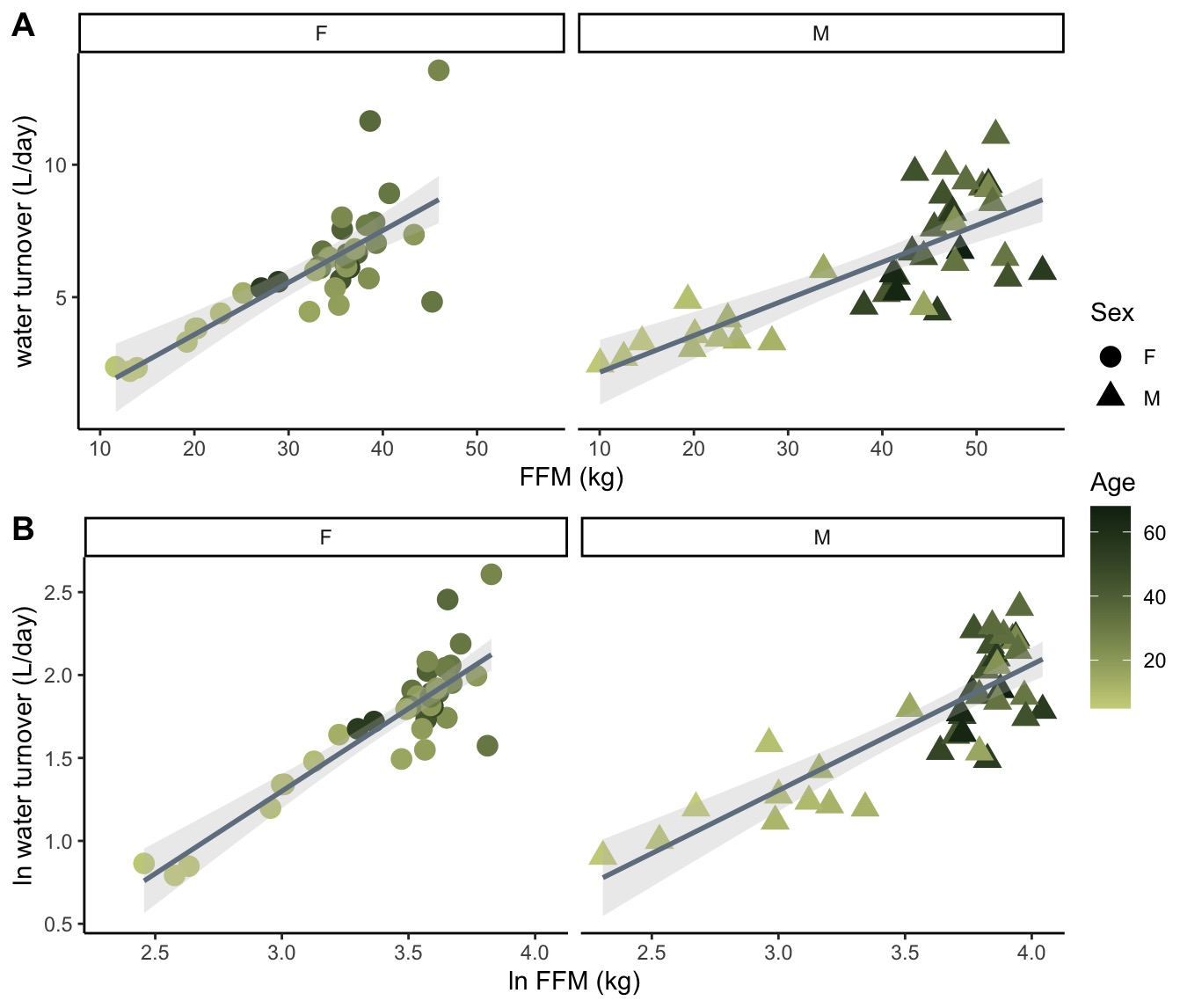


**Figure S1.** Water turnover (untransformed panel A; natural-log transformed, panel B) is positively correlated with fat-free mass (FFM) among Daasanach of both sexes (n=75).


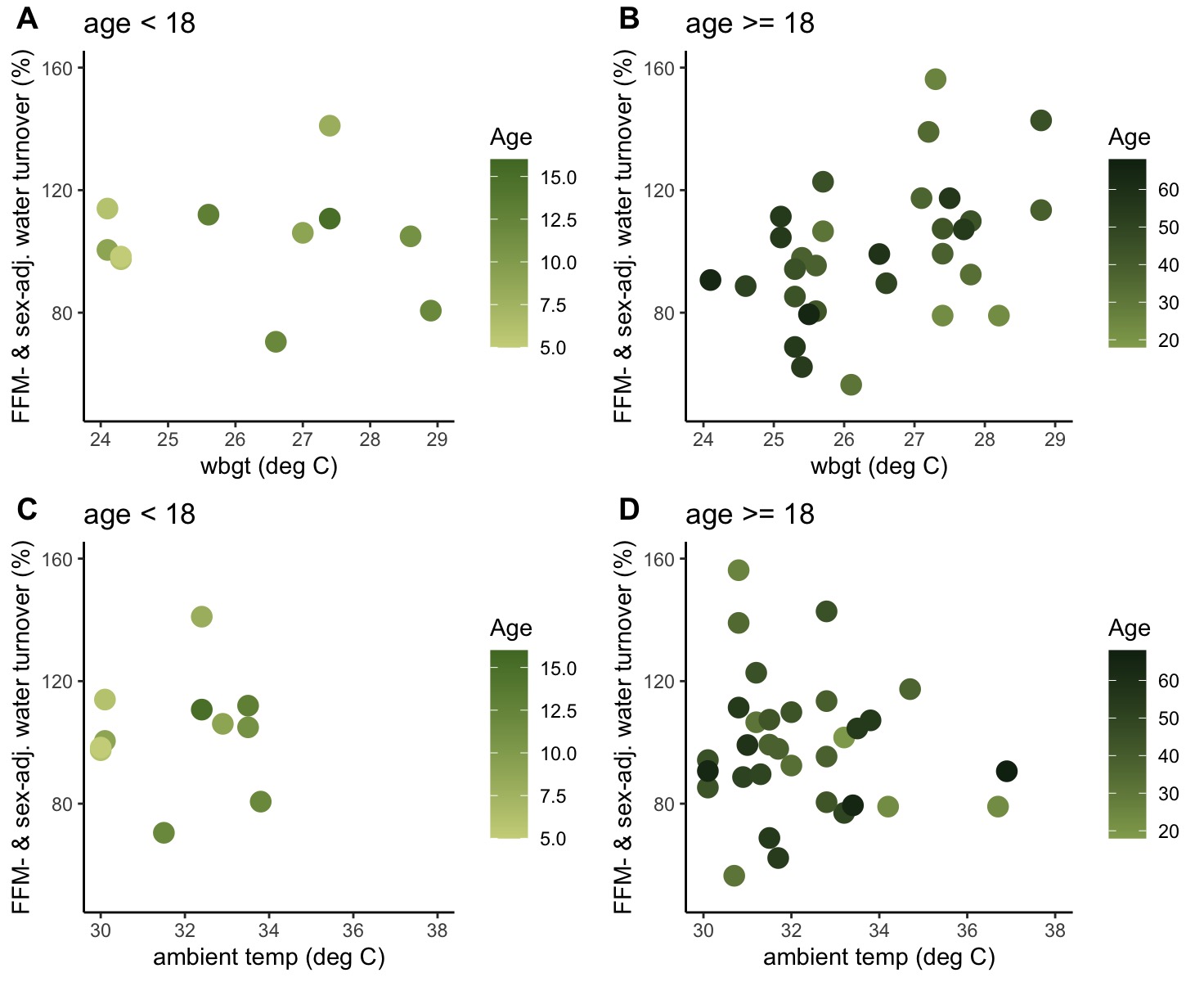


**Figure S2.** Fat-free-mass (FFM) and sex-adjusted water turnover as a function of wet bulb globe temperature (WGBT; panels A, B) and ambient temperature (panels C, D) for children (age<18, n=11) and adults (age≥18, n=36) at baseline urine collection. Water turnover was not significantly correlated with temperature when accounting for FFM and sex (p>0.05).


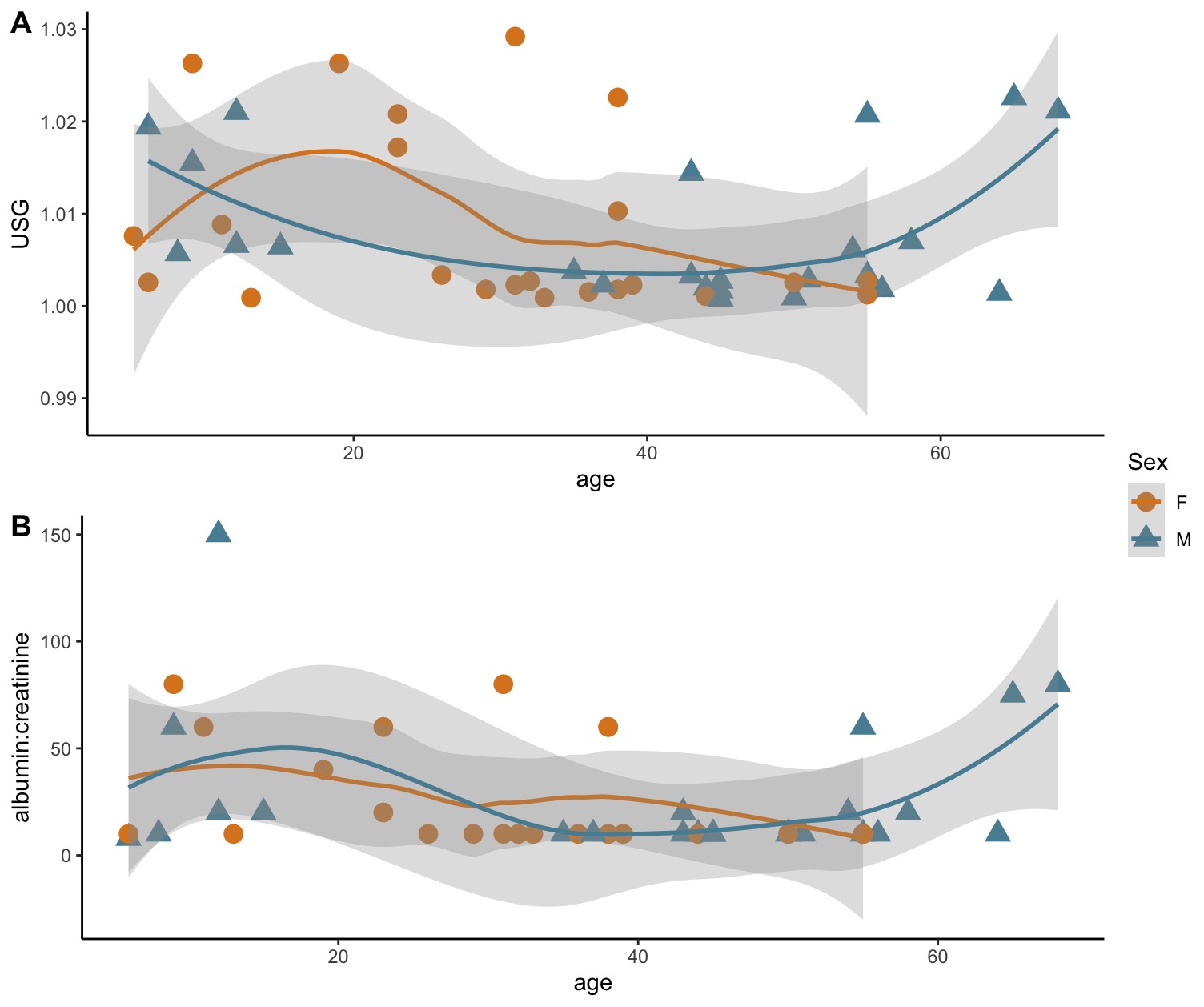


**Figure S3.** USG peaks early and late in life for male participants, but around age 20-30 for female participants; no differences in albumin:creatinine ratio (ACR) were noted between the sexes. ACR increases in men after age 50; we have no data for women at the oldest ages.


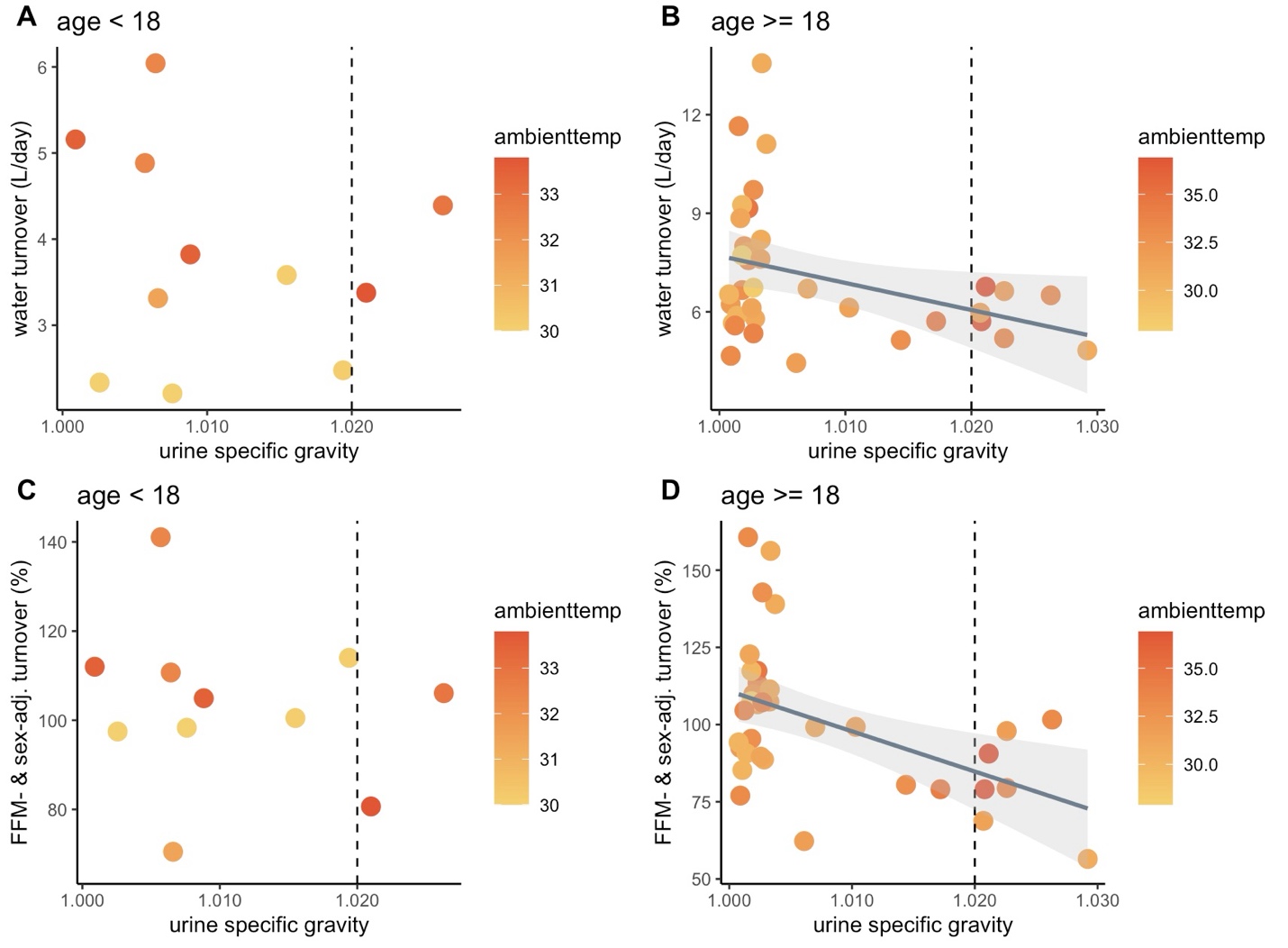


**Figure S4.** Absolute water turnover (A,B) and fat-free-mass (FFM) and sex-adjusted water turnover (B,D) as function of urine specific gravity and ambient temperature for children (age<18, n=11) and adults (age≥18, n=36). Dotted vertical line at USG = 1.020 represents the cut-off for dehydration, USG > 1.020 is considered dehydrated.


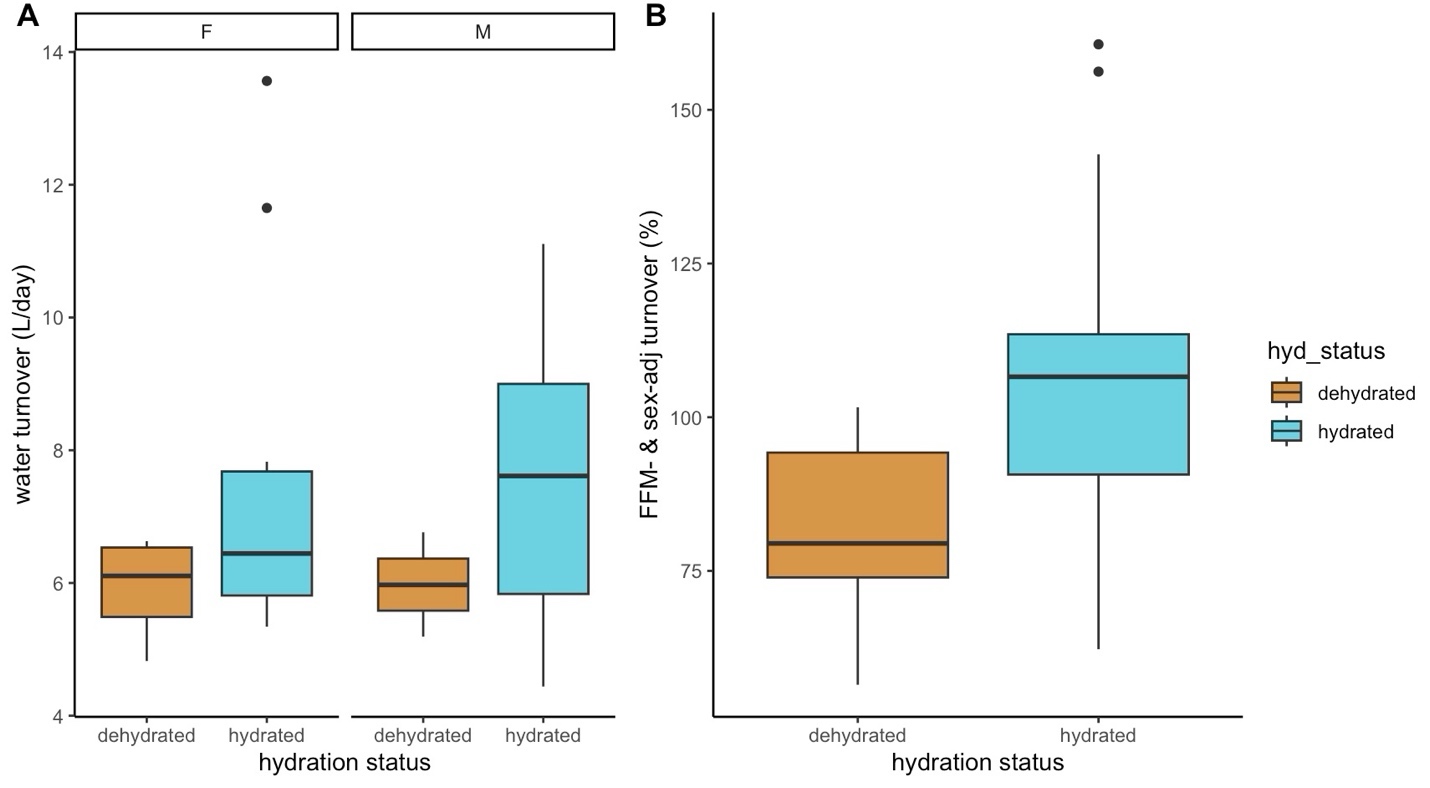
**Figure S5.** Absolute water turnover (panel A) and fat-free-mass (FFM) and sex-adjusted water turnover (panel B) by hydration status among adults (age≥18, n=36). Dehydrated individuals have a USG>1.020; hydrated individuals have USG ≤1.020.

**
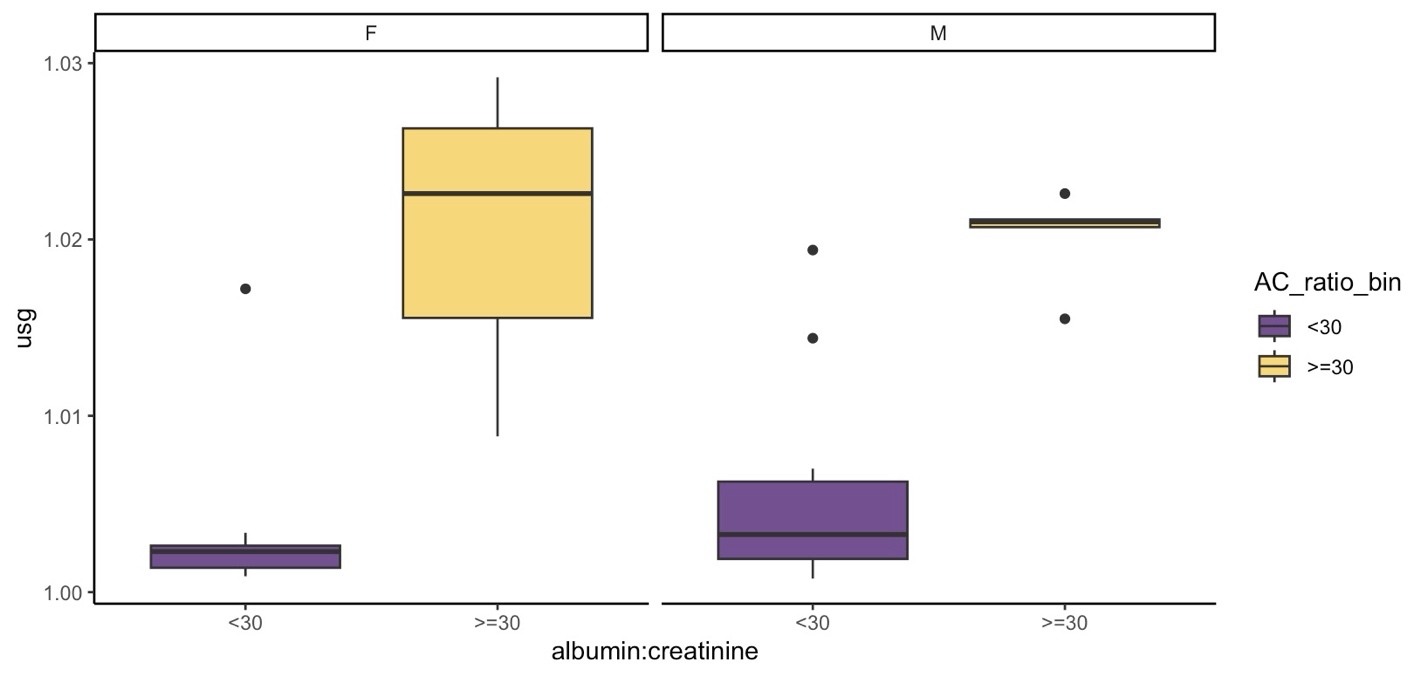
**

**Figure S6.** Urine specific gravity (usg) by albumin:creatinine ratio for all women and girls (F) and men and boys (M) (n=47).
